# Serologic Evidence for Novel Poxvirus in Endangered Red Colobus Monkeys, Western Uganda

**DOI:** 10.3201/eid1405.071686

**Published:** 2008-05

**Authors:** Tony L. Goldberg, Colin A. Chapman, Kenneth Cameron, Tania Saj, William B. Karesh, Nathan D. Wolfe, Scott W. Wong, Melissa E. Dubois, Mark K. Slifka

**Affiliations:** * University of Illinois, Urbana, Illinois, USA; † McGill University, Montreal, Quebec, Canada; ‡ Wildlife Conservation Society, Bronx, New York, USA; § University of California, Los Angeles, California, USA; ¶ Oregon Health and Science University, Portland, Oregon, USA

**Keywords:** Poxviridae, Orthopoxvirus, monkeypox, tanapox, Africa, Uganda, primates, colobus, serology, dispatch

## Abstract

Enzyme-linked immunosorbent assay, Western blot, and virus neutralization assays indicated that red colobus monkeys in Kibale National Park, western Uganda, had antibodies to a virus that was similar, but not identical, to known orthopoxviruses. The presence of a novel poxvirus in this endangered primate raises public health and conservation concerns.

The virus subfamily *Chordopoxviridae* contains emerging pathogens of considerable concern, both because of their historic impact on global human health and because of their zoonotic potential (*1*). Although certain poxviruses in this subfamily are among the most extensively studied viral pathogens (e.g., smallpox, vaccinia), the natural diversity of mammalian poxviruses remains poorly characterized. This fact is especially true in sub-Saharan Africa, where at least 1 zoonotic orthopoxvirus (monkeypox) has caused sporadic human outbreaks since 1970 (*2*), and infected rodents exported to the United States caused a highly publicized human outbreak in 2003 (*3**,**4*). In this study, we describe serologic evidence for a previously uncharacterized poxvirus in endangered red colobus monkeys (*Procolobus rufomitratus tephrosceles*) from Kibale National Park, western Uganda (*5**,**6*). Our results, based on postadsorption ELISA, Western blot, and virus neutralization assays, extend our understanding of the host range and diversity of poxviruses and raise public health and conservation concerns.

## The Study

From June 12 to June 24, 2006, 31 red colobus (13 males, 18 females, all adult or subadult) were sampled from Kibale National Park, western Uganda (*5**,**6*). Animals were chemically immobilized in the field by intramuscular injection of tiletamine/zolezepam (4.6–9.6 mg/kg body weight) with 1.0- or 1.5-mL plastic darts with 5/8-inch needles shot from a variable-pressure compressed air rifle. After initial darting, animals were given tiletamine/zolezepam or ketamine HCl intravenously as needed. Blood samples were collected into vacutainers containing sodium-EDTA, plasma was separated in the field by centrifugation, and samples were stored in liquid nitrogen for transport to North America. Animals were placed in cloth bags to recover from anesthesia and were released near trees and vines that were easy to climb and within visual range of their social groups. All animals appeared healthy at the time of capture. Animal protocols were approved by the McGill University Animal Care Committee before data collection.

A vaccinia virus (VV) ELISA was used as an initial screening test to detect antipoxvirus antibodies. VV-reactive antibodies were detected with horseradish peroxidase (HRP)–conjugated polyclonal goat anti–rhesus macaque (RM; *Macaca mulatta*) immunoglobulin (Ig) G (Fc specific; Nordic Laboratories, Tilsbug, the Netherlands), which readily detected antibodies of other primate species, including red colobus monkeys and humans. By using this approach, samples from 8 (26%) of 31 red colobus plasma scored positive (>200 ELISA units [EU]), 21 (68%) of 31 scored seronegative (<100 EU), and 2 (6%) of 31 had equivocal results (100–200 EU) (Appendix Figure).

**Figure 1:**
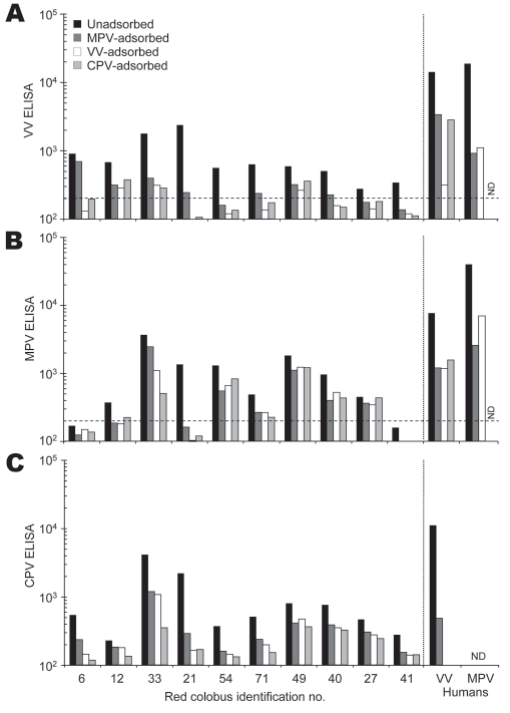
Serologic characterization of red colobus to *Orthopoxvirus* antigens. Plasma samples were collected from 31 red colobus, and 10 samples with detectable antibody responses to vaccinia virus (VV) antigens (Appendix Figure) were chosen for further analysis. Plasma samples were tested for specificity by a postadsorption ELISA (*7*) in which samples were either unadsorbed or preadsorbed with monkeypox virus (MPV), vaccinia virus (VV), or cowpox virus (CPV) antigens prior to performing an ELISA on A) VV-, B) MPV-, or C) CPV-coated ELISA plates. The results obtained by using plasma from a VV-immune human study participant (VV human) and a MPV-immune participant (MPV human) are shown for comparison. The dashed line indicates the cut-off value for a seropositive antibody response (200 ELISA units). ND, not determined.

Postadsorption ELISA tests (*7*) were used next to determine the specificity of the response to 1 of 3 orthopoxviruses: VV, monkeypox virus (MPV), or cowpox virus (CPV). Plasma samples were tested directly by ELISA or preadsorbed (1:30) for 30 minutes at 37°C with VV, MPV, or CPV lysate normalized to contain 6 × 10^8^ PFU/mL before addition to virus-coated ELISA plates (Figure 1). The postadsorption ELISA test allows one to differentiate among closely related orthopoxviruses. For instance, VV-specific antibodies are best depleted by VV lysate assayed on a VV-coated ELISA plate (Figure 1, panel** A**). However, analysis of samples from 10 red colobus with detectable anti-VV antibody responses (Appendix Figure) did not show a clear pattern following preadsorption with VV, MPV, or CPV antigens, which suggests a similar cross-reactivity to each of these 3 orthopoxviruses.

**Figure 2:**
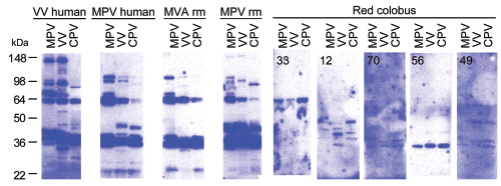
Western blot analysis of *Orthopoxvirus* (OPV)–reactive antibody responses in red colobus. Western blot analysis was performed to further characterize humoral immune responses against OPV antigens. Purified monkeypox virus (MPV), vaccinia virus (VV), and cowpox virus (CPV) were separated by sodium dodecyl sulfate-polyacrylamide gel electrophoresis, transferred to polyvinylidene difluoride membranes, and probed with plasma from a VV-immune human, MPV-immune human, MVA-immune RM, MPV-immune RM, and 5 representative red colobus. The red colobus animal identification number is shown in the upper left corner of each Western blot for comparison with the ELISA data for the same sample described in Figure 1.

Western blot analysis was performed to characterize more fully the antipoxvirus response of the red colobus. Two micrograms of purified MPV, VV, and CPV viral proteins were separated by 4%–20% gradient sodium dodecyl sulfate–polyacrylamide gel electrophoresis, transferred to polyvinylidene difluoride membranes (Invitrogen, Carlsbad, CA, USA), and probed with plasma (1:10,000) before addition of HRP-conjugated polyclonal goat antihuman Ig G (γ-specific, Jackson Immunolabs, Inc., West Grove, PA, USA) and chemiluminescent detection (Pierce SuperSignal West Dura Substrate, Rockville, IL, USA) (Figure 2). VV-immune human, MPV-immune human, MVA (modified vaccinia Ankara)-immune RM and MPV-immune RM plasma samples were included as positive controls to identify banding patterns typically observed in orthopoxvirus-immune humans and nonhuman primates. Western blot analysis proved more sensitive than anti-VV ELISA (Appendix Figure), with plasma from 30 of 31 red colobus reacting with at least 1 protein band from MPV, VV, or CPV. However, unlike the orthopoxvirus-immune human and RM controls, samples from red colobus demonstrated fewer immunoreactive bands and different immunodominant banding patterns, suggesting infection with either a distantly related orthopoxvirus or a virus from a different genus in the *Poxviridae* family.

Members of the *Orthopoxvirus* genus elicit cross-neutralizing antibodies against other members of the same genus. To determine if the red colobus were infected with an orthopoxvirus or a more distantly related poxvirus, plaque reduction neutralization assays were performed using 100 PFU of MPV, VV, or CPV. Plasma samples from all 31 red colobus were tested, and all exhibited a neutralizing titer 50 of <20 against MPV, VV, or CPV (data not shown). These findings suggest that although the monkeys were infected with a poxvirus with serologic cross-reactivity to VV (Appendix Figure), lack of a detectable neutralizing antibody response (<20) indicates that the animals may have been infected with a poxvirus that is not a member of the *Orthopoxvirus* genus.

## Conclusions

Our results provide evidence that red colobus in Kibale National Park have been exposed to a previously uncharacterized poxvirus. Kibale red colobus may have been exposed to monkeypox or to a “monkeypox-like” virus, but we could not confirm this with our current serologic tools. On the other hand, other poxviruses, such as Tanapox virus and Yaba monkey tumor virus, have been identified in Africa, and infection by 1 of these poxviruses or a related virus cannot be ruled out. Future studies will require optimizing serologic diagnostics against these divergent poxviruses (with appropriate positive and negative controls) to determine the identity of the poxvirus/poxviruses that have infected the red colobus described here. In this light, we note that tanapox, a zoonosis of suspected primate origin (*8*,*9*), derives its name from the Tana River, eastern Kenya, which supports an isolated population of red colobus closely related to those in Kibale (*P. r. rufomitratus*) (*10*,*11*).

A protracted outbreak of infectious disease occurred in Kibale red colobus from 1971 to 1981, where it caused a death rate up to nearly 10% in some social groups, apparently killing only adult male monkeys (*12*). Although neither formal clinical data nor biologic samples were collected, descriptions of lesions of affected monkeys suggested diffuse to multifocal areas of inflammation with gray mottling and epidermal crusts on the face (most commonly the lips), perineum, and inguinum, followed by alopecia and impaired locomotion. Monkeys sampled for the present study would almost certainly not yet have been born during this period, but these observations raise the possibility that outbreaks of disease at least outwardly consistent with poxvirus infection have occurred previously in Kibale red colobus.

Poxviruses are known for their potential to cross species barriers (*1*), and red colobus living in small, unprotected forest fragments outside of Kibale National Park interact aggressively and at high rates with local persons and their domestic animals (*13*). At the same time, persons in rural western Uganda already bear a high incidence of pathogens, including HIV (*14*), which renders a substantial proportion of the population immunocompromised and susceptible to opportunistic infections. Recent outbreaks of zoonotic poxviruses have not been documented in our study area, despite a local and regional healthcare system that would most likely have detected such events. However, the presence of a novel and potentially zoonotic poxvirus in red colobus should be viewed as a point of concern for the future of public health in this region and elsewhere.

## Supplementary Material

Appendix Figure Detection of Orthopoxvirus (OPV)-reactive antibodies in red colobus. Vaccinia virus (VV)-coated ELISA plates were used to test for antipoxvirus antibodies by endpoint dilution analysis as previously described (7). As positive controls, a representative VV-immune human (VV+ human) at 1 y postvaccination with DryVax (Wyeth Pharmaceuticals, Madison, NJ) and a modified vaccinia Ankara (MVA)-immune rhesus macaque (MVA+ RM) at 2 months post-vaccination with MVA are included for comparison. Negative controls included 2 unvaccinated human participants (OPV- human) and 6 unvaccinated rhesus macaques (OPV- RM). The dashed line indicates the cut-off value for a seropositive antibody response (200 ELISA units).
