# Supplementary figures and images for: Serologic Evidence for Novel Poxvirus in Endangered Red Colobus Monkeys, Western Uganda
Source: Emerg Infect Dis. 2008 May;14(5):801–3. doi: 10.3201/eid1405.071686 (PMC2600227; doi:10.3201/eid1405.071686)

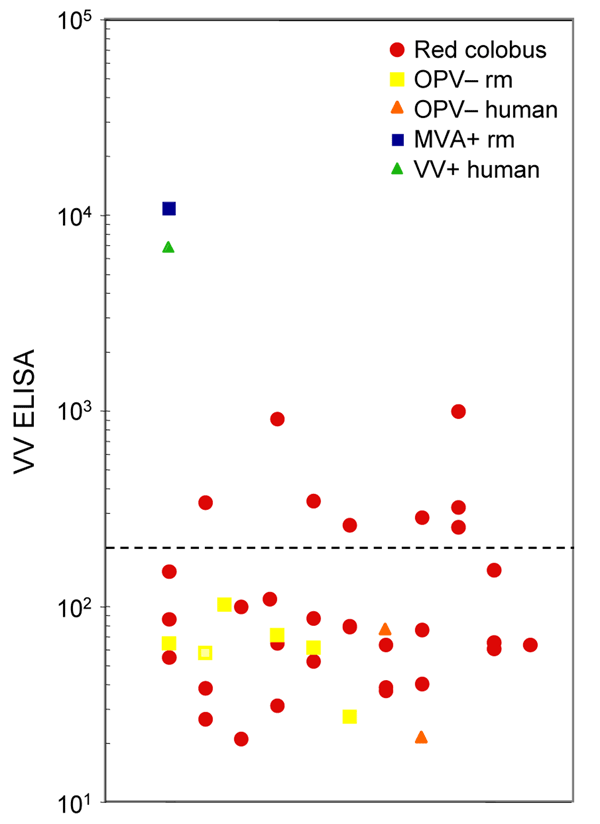

Supplement: Appendix Figure — Detection of Orthopoxvirus (OPV)-reactive antibodies in red colobus. Vaccinia virus (VV)-coated ELISA plates were used to test for antipoxvirus antibodies by endpoint dilution analysis as previously described (7). As positive controls, a representative VV-immune human (VV+ human) at 1 y postvaccination with DryVax (Wyeth Pharmaceuticals, Madison, NJ) and a modified vaccinia Ankara (MVA)-immune rhesus macaque (MVA+ RM) at 2 months post-vaccination with MVA are included for comparison. Negative controls included 2 unvaccinated human participants (OPV- human) and 6 unvaccinated rhesus macaques (OPV- RM). The dashed line indicates the cut-off value for a seropositive antibody response (200 ELISA units). [file 07-1686_app-s1.gif]
